# Advanced treatment of effluent extended aeration process using biological aerated filter (BAF) with natural media: modification in media, design and backwashing process

**DOI:** 10.1186/s13568-021-01260-2

**Published:** 2021-07-05

**Authors:** Mohammad Malakootian, Ali Toolabi, Saeed Hosseini

**Affiliations:** 1 Environmental Health Engineering Research Center, Kerman University of Medical Sciences, Kerman, Iran; 2 Department of Environmental Health Engineering, Faculty of Public Health, Kerman University of Medical Sciences, Kerman, Iran; 3 Department of Environmental Health Engineering, School of Public Health, Bam University of Medical Sciences, Bam, Iran

**Keywords:** Hospital wastewaters, Natural media, Biological aerated filters, Backwashing

## Abstract

Biological aerated filters (BAFs) have high filtration efficiency due to their tolerance of hydraulic and organic shocks are suitable for the treatment of complex and sanitary wastewater. In this study, for the first time, natural media of date kernel from Bam city was used as the BAF reactor media, with a meshing sand filter separated by a standard metal grid from the natural filter section used at the end of the reactor. This can be considered an innovation in the media and filtration. Aeration in the related reactor with 160 cm height was performed bilaterally as up-flow and continuously by nozzles throughout the reactor media. In this work, the actual effluent of the hospital wastewater treatment plant was employed as the inflow wastewater to the reactor, and its organic and inorganic parameters were measured before and after the treatment by the BAF reactor. The backwashing process was also studied in three ways: bottom backwashing (TB), top backwashing (BB), and top and bottom backwashing (TBBS), to determine the amount of water consumed and to achieve the desired result. According to the results obtained in this study, the removal efficiencies of inorganic and microbial contaminants, amoxicillin and azithromycin were obtained as follows: BOD_5_: 98.48%, COD: 92.42%, ${\text{NO}}_{3}^{ - }$: 99.4%, P: 93.3%, Coliforms: 97%, Color: 42.8%, Turbidity: 95%, Sulphate: 30%, TSS: 98.9%, Amoxicillin: 20% and azithromycin: 13%. In the backwashing process, the amount of water consumed in these three TB, BB, and TBBS methods were obtained 300, 164, and 118 L, respectively, So, TBBS method was selected as the optimal method. Based on the results obtained in this study, it is concluded that the BAF process with natural date kernel has a high efficiency in removing organic and inorganic contaminants from hospital wastewater, also the concentration of most of the effluent parameters was less or in accordance with EPA standard.

## Introduction

Hospital wastewaters (HWW) are considered an important source of water contamination. These wastewaters contain large amounts of dangerous pollutants including pathogens, fats, proteins, carbohydrates, pharmaceuticals, resistant chemicals, and endocrine disrupters (Zou 2015; Liu et al. 2010). The concentration of micro-pollutants existing in these wastewaters is 4–150 times higher than that in urban wastewaters (Kovalova et al. 2013). Some components of hospital wastewater are genotoxic and carcinogenic to humans (Gurjar et al. 2019). These wastewaters can enter water sources, sediments, and soil through incomplete wastewater treatment systems (Huang et al. 2011; Gros et al. 2007, Navasero and Oatman 1989; Aboltina et al. 2017). Thus, due to the existence of diverse and complex compounds in hospital wastewaters, conventional wastewater treatment plants cannot completely remove them and advanced treatment methods should be used (Aboltina et al. 2017; Verlicchi et al. 2012; Nikoonahad et al. 2017). Biological aerated filters (BAFs) are a novel, flexible, and economical method with low footprint in the formation of active microbial biofilms and high organic charge (Lee et al. 2013; Liu et al. 2015; Antoniou et al. 2013; Bohm 2007; Shi et al. 2011). These filters are used as a desirable biological process for treating waste leachates, waste-containing pathogens, volatile organic compounds, nitrate, ammonium, phosphorus, suspended solids, acetate, fats, dyes, and nitro-aromatic compounds (Shi et al. 2011; Gehr et al. 2003; Sheng et al. 2010; Xu et al. 2012; Tudor and Lavric 2011). Filtration, absorption and biodegradation are important mechanisms of the BAF process in wastewater treatment (Nikoonahad et al. 2018).

The most important materials used in BAF media are sand, concrete, clay, shale, polyethylene, polystyrene, and plastic materials (Abouelela et al. 2019; Bilotta and Brazier 2008). The diameter of these media is about 4 mm, operating at 10 h hydraulic retention time (Ding et al. 2018; Blumenthal et al. 2000; Amin et al. 2018). Nikoonahad in 2017 used a BAF reactor with a modified polystyrene medium in Iran, which was coated with sand as a new medium for advanced domestic wastewater treatment (Nikoonahad et al. 2017). Liu Jianguang in China treated hospital wastewater using BAF method in 2003 (Jianguang 2003). YI Biao et al. in 2007 treated urban wastewater applying a BAF reactor and achieved 94.7% BOD removal (Biao et al. 2007).

City of Bam in Kerman Province is located in southeastern Iran with the average rainfall of 56.7 mm per year, and producing 200,000 tons of dates. Date is a fruit from *Phoenix dactylifera* family. Date kernel has a length between 2.5 and 2.8 cm, width of 0.8–0.9 cm, and thickness of 0.5 to 0.6 cm (Zayed and Eisa 2014). Date kernel contains a large amount of nutrients for the growth of microorganisms (Besbes et al. 2004a).

In a study in Tunisia by Besbes et al. (2004b), chemical and physical properties of date kernel were analyzed. It was found that date kernel contains high amounts of protein and unsaturated fats such as oleic acid and carbohydrates, which are essential for the growth of living creatures (Besbes et al. 2004b). In Saudi Arabia, Al-Thubiani in 2017 evaluated date kernel powder as a probiotic material containing large amounts of *lactobacillus paracasei* (Althubiani and Ahmadkhan 2017). Thus, due to the presence of these nutrients in date kernel, in the present study, applicability of biological aerated filter (BAF) with natural date kernel media in the removal of nitrate, phosphate, total suspended solids (TSS), biological oxidation demand (BOD), chemical oxygen demand (COD), Color, sulphate, coliforms, turbidity, azithromycin and amoxicillin from the outflow effluents of Pastor Hospital in Bam city were evaluated. The research innovations include the use of natural date kernel media instead of synthetic media (such as polystyrene), do not need to add nutrients for biofilm growth due to the use of natural date kernel media, which is known to have nutrients for the growth of microorganisms, the use of BAF reactor with natural date kernel in hospital wastewater treatment as the first study done so far, obtaining outflow effluent from a very high quality reactor and the use of different media in the backwashing process.

## Materials and methods

### Sampling and wastewater features

In this study, Sampling of raw wastewater and effluent from wastewater treatment plant of Pastor Hospital in Bam city, Iran was performed. After sampling, the samples were transferred to a specialized laboratory for analysis. Based on the results obtained from raw wastewater, the amount of BOD, COD, TSS, nitrate, sulfate and phosphate were reported 320, 440, 970, 76, 1050 and 45 mg/L, respectively. Also the amount of amoxicillin and azithromycin in raw wastewater were obtained 15 and 3, respectively. Other characteristics of raw wastewater and effluent are shown in Table 1.

**Table 1:** Characteristics of raw wastewater and effluent from wastewater treatment plant

Parameter	Mean raw wastewater	EPA standards	Mean effluent
pH	7.8	6–9	8.1
Temperature	20 ± 19	–	2 ± 18
TSS (mg/L)	15 ± 970	5	5 ± 90
DO (mg/L)	20 ± 0.75	2	2 ± 2
BOD_5_ (mg/L)	20 ± 320	10	15 ± 100
COD (mg/L)	25 ± 440	100	15 ± 198
${\text{NO}}_{3}^{ - }$ (mg/L)	10 ± 76	50	5 ± 30
${\text{PO}}_{4}^{ - }$ (mg/L)	12 ± 45	6	5 ± 30
Turbidity (NTU)	–	50	2 ± 10
Color (TCU)	45 ± 890	550	5 ± 105
Sulfate (mg/L)	57 ± 1050	1500	20 ± 600
Coliforms (MPN)	1000 ± 1 × 10^7^	1000	100 ± 1660
Amoxicillin (mg/L)	3 ± 15	–	3 ± 10
Azithromycin (mg/L)	20 ± 3	–	0.3 ± 1.5

### Reactor construction and operation

**Fig. 1:**
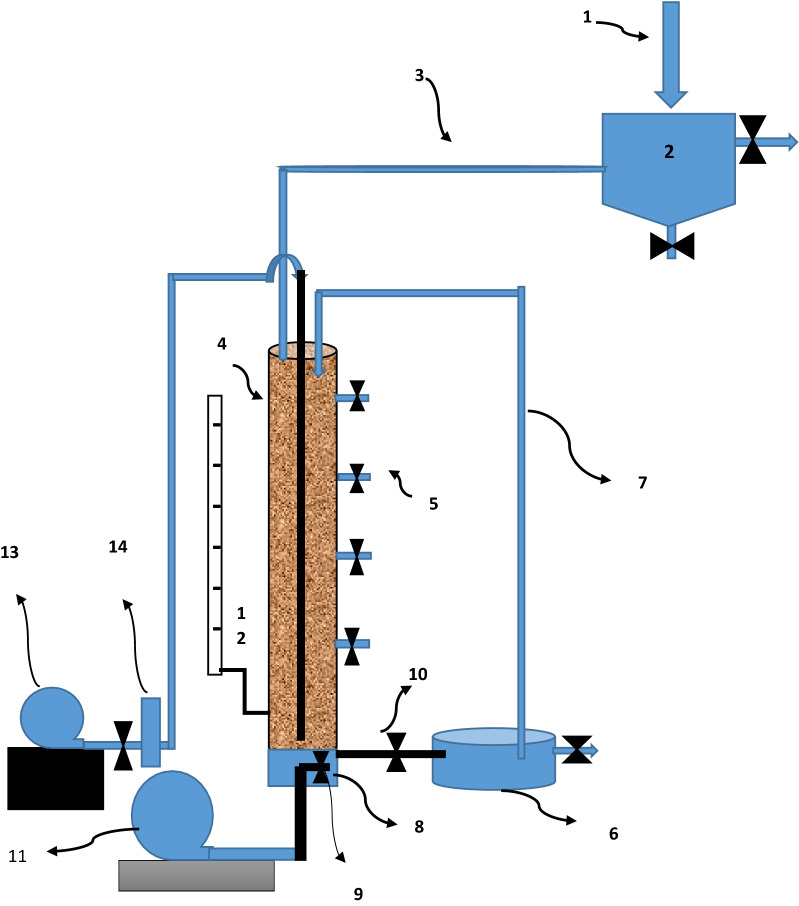
Schematic of the BAF reactor (1. Wastewater Inlet 2. Primary settling tank 3. Wastewater inlet line 4. Reactor chamber 5. Sample valves 6. Secondary settling tank 7. Return sludge line 8. Sand filter 9. One-way valve 10. Effluent Valve 11. Backwash pump 12. Piezometer 13. Aeration pump 14. Aeration regulator)

In this study, a biological aerated filters (BAFs) reactor was used to treatment the effluent from the wastewater treatment plant. The hydraulic properties of the designed reactor are shown in Table 2. A cylindrical tube made of polyvinyl chloride with a height of 110 cm and inner diameter of 10 cm filled to the height of 60 cm from the date kernel natural media was used to fabricate the reactor (Fig. 1). The date kernel medium is a natural media containing sufficient amounts of date residues for the initial feeding of microorganisms (Chandrasekaran and Bahkali 2013).

**Table 2:** Reactor hydraulic properties

Parameter	Unit	Value
Hydraulic retention time	min	60
Wastewater flow rate	L/m^2^ s	0.22
Back wash flow rate	L/m^2^ s	1.6
Flow rate of aeration pump	L/m^2^ s	0.9
Reverse aeration pump flow rate	L/m^2^ s	7.3
Average organic loading	Kg COD/m^3^ d	0.76
Mean dissolved oxygen	mg/L	5
Inlet discharge to the reactor	mL/L	100

In order for maintenance of the equilibrium of the main media and prevention of possible biofilm particle outflow, three clay layers with 0.2, 0.4, and 0.6 mm meshing and 20 cm height were placed, where the bottom of the reactor was separated by a metal filter of stainless steel in 0.1 mm diameter from the above section of the reactor. The inflow to the reactor was used as up-flow to save and facilitate energy. To review the impact of the reactor height on removal of the desired pollutants, a recovery valve was inserted every 25 cm from the tube length.

In order to check the pressure inside the reactor, a piezometer was used in it. The aeration was performed continuously by an external pump with the discharge of 0.3–0.9 L/m^2^ s. To wash the filter media and prevent filter obstruction as well as outflow of additional biomass from the bottom of the reactor, backwashing was performed by an air pump with discharge of 8 L/m^2^ s every 20 days for 20 min. To avoid turbulence in the sand filter, the backwashing pump hose was connected to a one-way valve mounted in the middle of the stainless-steel filter. A 100-L tank was employed for balancing and for initial sedimentation of the effluent entering the reactor. The effluent in the tank was gravity-fed into a biological reactor by a number of control valves. To reach the end of the backwashing, the outflow effluent turbidity was measured continuously. Other specifications are listed in Table 2.

### Analytical methods

For biological adaptation and biofilm formation on the date kernel media, the outflow effluent from the hospital was aerated on a BAF filter for 4 weeks. After biofilm formation, the surface morphology of biofilm was performed by Field Emission Scanning Electron Microscopes (FESEM), the outflow of actual hospital treatment plant effluent was injected continuously at 100 mL/min discharge from the top of the reactor. After satisfying the above conditions, all parameters of this study including; pH, temperature, dissolved oxygen, oxidation potential, and analysis reduction were analyzed by multi-parameter HANNA (model, HI98196, made in Italy) (Romero et al. 2008), while turbidity was measured by HACH portable turbidimeter (model 2100Q). BOD_5_ was also measured by BOD 6-chamber device (BOD Oxidirect manufactured by Lavi band company, Germany) (Beszédes et al. 2018).

COD was measured through standard reflux method (Vyrides and Stuckey 2009); and azithromycin and amoxicillin antibiotics were analyzed using high-performance liquid chromatography device (HPLC) as well as C18 specific column, mobile phase (95% phosphate buffer and 5% acetonitrile) (Cazorla-Reyes et al. 2014). TSS was analyzed via gravimetric method (Alkarkhi et al. 2008), coliforms (based on the most probable number of coliforms per 100 cc) (Evans et al. 1981), as well as nitrate and phosphate according to the procedures addressed in Water and Wastewater Standard Method. Eddy. “eddition”, 23 (2017) (Metcalf et al. 1979). Based on Eq. (1), the efficiency of the BAF process in removing pollutants was obtained.

1 $$Removal\left(\mathrm{\%}\right)=\left(\frac{C0-Ct}{C0}\right)\mathrm{*}100,$$

where: C_0,_ initial concentration of pollutant (mg/L); C_t_, residual concentration of pollutant (mg/L).

### Backwashing process

The backwashing process was performed based on the pressure drop in the piezometer between 13 and 17 cm. Initially, the backwashing pump was turned on for 20 min and, during the washing, the effluent from the turbidity was evaluated until the desired turbidity was reached. The backwashing process was also studied in three ways: bottom backwashing (TB), top backwashing (BB), and top and bottom backwashing (TBBS), to determine the amount of water consumed and achieve the desired result. In all cases, the outflow turbidity was evaluated from the release valves. In the TBBS technique, backwash effluent flow rate through bottom and top valves was approximately 70% and 30%, respectively. During steps one and two totally, nine backwashes were conducted in TB, BB, and TBBS methods; each one for three times. In each backwashing process, the air compressor with an air flow rate of 8 L/m^2^ s was initially applied for 20 min to dislodge the solid germs and biological slug mass by creating turbulence in the media and silica layer. Then, the air flow was shut down and immediately clean water with a flow rate of 1.6 L/m^2^.s was applied from the same entrance to separate and drive the BAF sludge out.

### Preparation and analysis of date kernel

Nutrient tests were performed to investigate the nutrients and other organic and inorganic constituents of the date kernel to be used as a natural substrate for the BAF reactor. The date kernels were firstly isolated and then collected from the palm mantle, and after repeated washing with warm water, the final rinse was deionized. Afterward, they were kept under the sun light for 2 days. Subsequently, they were dehydrated for 24 h at 50 °C. The cores were then powdered through the mill and dissolved in 100 mL of chloroform and acetone and finally allowed to stand for 48 h at room temperature. After passing the homogeneous solution of Whitman filter, physicochemical and Brunauer–Emmett–Teller (BET) analyses were determined (Platat et al. 2014; Baliga et al. 2011). Mineral content [N, K^+^, Ca^2+^, Na^+^] was determined by using the nitrate, potassium, calcium and sodium digital meter, model: Horiba (USA), Iron and manganese were determined using Atomic Absorption Spectrophotometer (Parvin et al. 2015; Sharif 2015).

### Statistical analysis

Data on pollutant removal efficiency were analyzed by SPSS (ver. 22) software through one-way analysis of variance test.

## Results

### The results of the physicochemical and Brunauer–Emmett–Teller (BET) properties tests of dates kernel

The results of the physicochemical properties of date kernel are shown in Table 3, and the results of date mineral analysis were as follows: sodium: 25.33, calcium 25.33, manganese 4.5, potassium 0.6 and iron 43.76 eq/L, which was the highest amount of mineral related to iron. Also, particle density and crude fiber content of date kernels were obtained 650 kg/m^3^ and 26.18%, respectively. The results of the Brunauer–Emmett–Teller (BET) properties of date kernel are shown in Table 4. According to the results obtained in Table 4, Total pore volume and mean pore diameter of date kernel were reported 0.0023731 cm^3^ g^−1^ and 9.4649 nm, respectively.

**Table 3:** Physico-chemical properties of date kernels

Parameter	Value
Ash content %	1.52
Moisture %	2.33
Volatile content %	65.4 ± 3.5
Fixed carbon content %	25 ± 3
C %	28
N %	0.8
Fat %	12
Particle density Kg/m^3^	650
Prosity %	24
Total carbohydrate %	79.33
Crude Fiber %	26.18
Protein %	4.44
pH	4.7 ± 0.3

**Table 4:** BET Plot of date kernel

Parameter	Value	Unit
*V*_*m*_	0.2304	[cm^3^(STP) g^−1^]
a_s,BET_	1.0029	[m^2^ g^−1^]
*C*	67.951	
Total pore volume (*p*/*p*_0_ = 0.990)	0.0023731	[cm^3^ g^−1^]
Mean pore diameter	9.4649	[nm]

### The field-emission scanning electron microscope of the date kernel

**Fig. 2:**
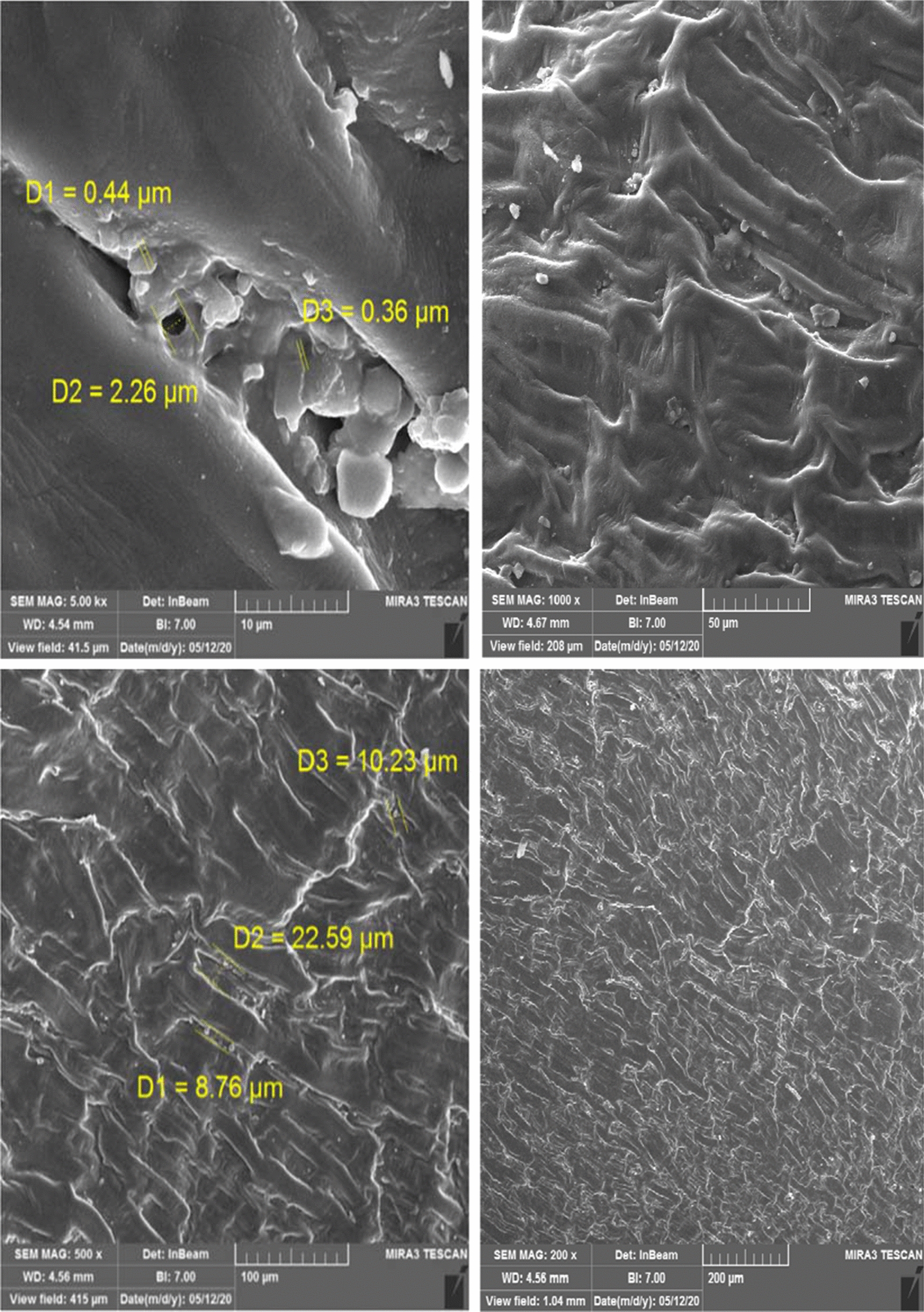
FESEM of date kernel media

The results of the field-emission scanning electron microscope (FESEM) of the biofilm formed on the date kernel are shown in Fig. 2. Date kernel surface morphology showed that the biofilm formed was very rough, bumps and protrusions. The size distribution of these protrusions was quite variable (range of 0.36–22.59 µm).

### The results of biological filtration in removal of pollutants

The results of biological filtration by date kernels are presented in Table 5. In this study, upon increasing the filtration height from 25 to 100 cm, the removal efficiencies of inorganic and microbial contaminants, amoxicillin and azithromycin were obtained as follows: BOD_5_: 98.48%, COD: 92.42%, ${\text{NO}}_{3}^{ - }$: 99.4%, P: 93.3%, Coliforms: 97%, Color: 42.8%, Turbidity: 95%, Sulphate: 30%, TSS: 98.9%, Amoxicillin: 20% and azithromycin: 13%.

**Table 5:** Results of biological filtration by date kernels

Parameter	Inlet wastewater to the reactor	Average output of the reactor at a height of 25 cm	Average output of the reactor at a height of 50 cm	Average output of the reactor at a height of 75 cm	Average output of the reactor at a height of 100 cm	Average output from drain valve reactor	Average final removal percentage
PH	8.1	8.1	8.1	8.1	0.5 ± 8.1	1 ± 7	–
DO (mg/L)	2	0.2 ± 5	0.3 ± 5	0.3 ± 5	0.3 ± 5	0.3 ± 5	–
${\text{BOD}}_{5}^{ - }$ (mg/L)	5 ± 100	5 ± 80	5 ± 50	5 ± 20	5 ± 10	0.6 ± 3	98.48
COD (mg/L)	10 ± 198	10 ± 150	7 ± 70	5 ± 30	5 ± 20	3 ± 15	92.42
${\text{NO}}_{3}^{ - }$ (mg/L)	5 ± 30	2 ± 20	1.5 ± 15	1 ± 8	0.5 ± 3	0.3 ± 0.6	99.4
${\text{PO}}_{4}^{ - }$ (mg/L)	3 ± 15	2 ± 10	1.5 ± 8	1 ± 4	0.8 ± 2	0.3 ± 1	93.3
Turbidity (NTU)	0.5 ± 10	0.5 ± 9	0.5 ± 8	0.5 ± 7	0.5 ± 6	0.5	95
Color (TCU)	10 ± 105	10 ± 100	10 ± 80	10 ± 75	10 ± 65	10 ± 60	42.8
Sulfate (mg/L)	80 ± 600	80 ± 580	80 ± 480	80 ± 450	60 ± 430	50 ± 420	30
TSS (mg/L)	0.2 ± 90	0.2 ± 40	0.2 ± 22	0.2 ± 15	0.2 ± 9	0.2 ± 1	98.9
ORP (mv)	+ 10	+ 50	+ 85	+ 120	+ 200	+ 214	–
Amoxicillin (mg/L)	3 ± 10	–	–	–	–	1 ± 8	20
Azithromycin (mg/L)	0.3 ± 1.5	–	–	–	–	0.05 ± 1.3	13

### Evaluating backwashing finishing time process

Based on the pressure drop in the piezometer, the backwashing process was performed every 20 days. According to the results obtained in this study, the TB backwashing method was completed in 75 min, while in the BB method, the optimum turbidity was completed in 45 min. In the TBBS method, the optimum turbidity removal rate was obtained within 20 min. The amount of water consumed in these three TB, BB, and TBBS methods was 300, 164, and 118 L, respectively. The lowest amount of water consumed for washing was related to the TBBS process.

### Evaluating effluent backwashing return to reactor

In this study, the effluent backwashing return to the reactor and its outflow was investigated over two three-month periods and the results of the pollutant analysis were compared with the reactor outflow without the return of effluent, Table 6. The lowest and highest COD removal efficiencies were related to Reactor output without return effluent and TBBS methods, respectively. The removal efficiency of other Parameters by the studied methods was slightly different.

**Table 6:** Properties of effluent backwashing return to the reactor

Method	BOD (mg/L)	COD (mg/L)	TSS(mg/L)	Turbidity (NTU)
Reactor output without return	3	15	1	0.5
Reactor output with TB rotation	2	13	1	0.5
Reactor output with BB rotation	2	13	1	0.5
Reactor output with TBBS rotation	2	12	1	0.5

## Discussion

The results of the field-emission scanning electron microscope of the biofilm formed on the date kernel are shown in Fig. 2. Date kernel surface morphology showed that the biofilm formed was very rough, bumps and protrusions. The size distribution of these protrusions was quite variable (range of 0.36–22.59 um). Based on the results obtained in this study, The FESEM technique showed that, Colony growth and biofilm formation on date kernels are higher than polymeric materials. Also, in the present study, the removal efficiency of contaminants from wastewater was higher than, other studies using polymeric and synthetic media (Nikoonahad et al. 2018). According to Nikoonahad et al. study in 2017, it has been reported that the rough and irregular surface of media has provided a suitable site for biological attached growth (Nikoonahad et al. 2017).

In this study, BET test was used to measure the amount of porosity and effective surfaces of the date bed, with its results shown in Table 4. Based on the results, it was observed that there was a direct relationship between the amount of bed pores and the amount of adsorption and decomposition of contaminants. According to the results presented in Table 5, the removal efficiencies of BOD_5_, COD, ${\text{NO}}_{3}^{ - }$, P, Total Coliforms, Color, Turbidity, Sulphate, TSS, Amoxicillin and azithromycin were obtained, 98.48, 92.42, 99.4, 93.3, 97, 42.8, 95, 30, 98.9, 20 and 13%, respectively. Thus, the concentration of pollutants in the effluent was observed in the range of environmental standards.

Liu et al. (2010) in China treated domestic wastewater by a BAF reactor with oyster shell media as well as plastic ball media. They achieved COD, PO_4_, and ${\text{NO}}_{3}^{ - }$ removal efficiency of 85.1%, 98.1%, and 79.9% for oyster shell media and 80%, 93.7%, and 90.6% for plastic ball media, respectively (Liu et al. 2010).

Abouelela et al. (2019) in Egypt used a BAF reactor to treat domestic wastewater and reached the BOD and COD removal efficiency of 92% and 89%, respectively (Abouelela et al. 2019), In the present study, it was found that the removal efficiency of contaminants was higher than other studies, the reason for which could be the use of natural date kernel media. Also, ORP was reported at lower + 50 mV depth and, with the increase in the reactor floor height, ORP levels were increased as well. In aerobic processes due to increased dehydrogenase enzyme activity, ORP levels also increased, improving and facilitating organic material degradation (Toolabi et al. 2017, 2018, 2019).

According to Rebecca’s Moore’s studies carried out in 2001 in the UK, as the media depth increased, so did the removal efficiency of parameters such as BOD, COD, and TSS due to longer retention time and greater opportunity for the degradation of pollutants by biofilm microorganisms (Moore et al. 2001). BAF reactors have both oxic and anoxic zones. Anoxic zones are suitable for nitrification and denitrification, removing organic matter and nitrate, as well as removing oxic residual zones of organic matter and ammonium (Pramanik et al. 2012). Phosphorus removal from wastewater is accomplished by sedimentation and absorption. So, in the current study we observed enhanced removal efficiency for all pollutants with increasing retention time and reactor length.

Given the high per capita production of dates in Bam, it is important to use natural date kernel media for secondary high-quality treatment of wastewater and effluent for discharge into receiving waters. Due to the interstices on the kernel and large amount of residual nutrients in the kernel shell, or so-called endocarp, it is a suitable environment for the growth of microorganisms decomposing organic matter of wastewater (Ravi 2017; Dehdivan and Panahi 2017; Platat et al. 2014; Baliga et al. 2011).In this study, according to the results obtained, date kernel contains a large amount of minerals such as calcium, magnesium, phosphorus, iron, zinc, and flavonoids. Similarly, in the experiments performed on Bam Mazafati date kernel by Dehdivan et al. (2017) in Iran, it was found that date kernels contained large amounts of organic and mineral nutrients (Dehdivan and Panahi 2017).

According to the results obtained in this study, the TB backwashing method was completed in 75 min, while in the BB method, the optimum turbidity was completed in 45 min. In the TBBS method, the optimum turbidity removal rate was obtained within 20 min. The amount of water consumed in these three TB, BB, and TBBS methods was 300, 164, and 118 L, respectively. The lowest amount of water consumed for washing was related to the TBBS process, consequently this method was selected as the optimal one. In the study by Nikoonahad (2017) on a BAF reactor with polystyrene media about backwashing media, TBBS method with 35 min was selected as an optimum method (Nikoonahad et al. 2017).

In this study, to prevent biofilm outflow, a three-layer sand grading filter was considered, which would further remove the turbidity of the outflow effluent from the reactor. The date kernel as a food source for the growth of microorganisms as well as the presence of viscous layer compounds on the date kernel slowed down the movement of wastewater, resulting in small water ponds throughout the reactor (Song et al. 2014). In addition, further meshing and degradation of pollutants were observed by biofilm.

In this work, the effluent backwashing return to the reactor and its outflow was investigated over two three-month periods. Then, the results of the pollutant analysis were compared with the reactor outflow without the return of effluent. According to the data in Table 6, slight changes were observed in the removal of pollutant parameters with the return of the backwash effluent to the BAF reactor due to the increased presence of microorganisms in the effluent. In aerobic wastewater treatment processes, the observation of *protozoan* and *metazoan* microorganisms is an indication of adapted microbial biofilm for the decomposition of wastewater organic matters (Kamika and Momba 2013). In wastewater treatment by the attached growth methods, we usually see the presence of *protozoa*, *metazoan*, *mastigamoeba, vorticella* and *rotifer* (Madoni 2011).

In this study, the removal efficiency of amoxicillin and azithromycin by BAF process were observed 20% and 13%, respectively. Antibiotics are not easily degraded via biological processes due to their complex structure and require complex processes such as photocatalytic processes as well as advanced oxidation for complete removal (Manaia et al. 2018). In this study, wastewater treatment was performed by aerated biological filters with modifications and innovations in media (date kernel) as well as three methods of backwashing and outflow effluent evaluation. Here, very favorable results were achieved in the removal of indicator pollutants as well as other physical and chemical parameters. Some of the results were concerned with the percentage of TSS (1 mg/L), turbidity (0.5 NTU), sulphate (420 mg/L), nitrate (0.18 mg/L), COD (14.9 mg/L), BOD_5_ (1.52 mg/L), and phosphate (2 mg/L), which were in accordance with EPA guidelines, as in Tables 1 and 5. Given that the natural medium of date kernels has a porous surface and contains nutrients for the growth of microorganisms, so, in comparison with other synthetic media, it never requires adding nutrients for the growth of microorganisms and biofilm formation. In the present study, it was found that the BAF reactor with natural media of date kernel via filtration, absorption and biodegradation mechanisms causes wastewater treatment. Effluent recirculation of backwashing in all the three methods of top, bottom and top–bottom backwashing, as compared in Table 6, showed no significant effect on the removal of pollutants in these three methods as well as effluent without recirculation method. In the present study, also, oxidation and reduction potential was measured in order to prevent anoxic media along the reactor at different heights. The results ranged from + 10 mV in raw wastewater to + 214 mV in outflow effluent, indicating optimum aeration performance. The proposed aeration biological filter system can be used as an effective method for secondary treatment of wastewater effluent.

## Data Availability

Not applicable.

## Abbreviations

HRT: Hydraulic retention time
BAF: Biological aerated filters
COD: Chemical oxygen demand
ORP: Oxidation reduction potential
HPLC: High performance liquid chromatography
HWW: Hospital waste waters
BB: Bottom backwashing
TB: Top backwashing
TBBS: Top and bottom backwashing
